# C9orf72 Alleviates DSS‑Induced Ulcerative Colitis via the cGAS‐STING Pathway

**DOI:** 10.1002/iid3.70139

**Published:** 2025-01-28

**Authors:** Yue Wang, Ting Xu, Wenjun Wang

**Affiliations:** ^1^ Department of Gastroenterology Qingdao Municipal Hospital Qingdao Shandong China; ^2^ Department of Health Care Qingdao Municipal Hospital Qingdao Shandong China

**Keywords:** C9orf72, cGAS‐STING pathway, ulcerative colitis

## Abstract

**Purpose:**

C9orf72 deficiency contributes to severe inflammation in mice. Ulcerative colitis (UC) is a chronic inflammatory disorder with the shortage of clinical success. However, whether C9orf72 is involved in the progression of UC is not fully understood. This study investigated whether C9orf72 could alleviate dextran sulfate sodium (DSS)‐induced colitis in mice and lipopolysaccharide (LPS)‐induced colitis in Caco‐2 cells.

**Methods:**

Mice were injected AAV9‐C9orf72 lentivirus through tail vein and fed 3% DSS for a week. Caco‐2 cells were cultured to establish C9orf723 overexpressed model. Histopathological examination, level of inflammation, cGAS‐STING pathway, and gut barrier function were detected in mice and cells.

**Results:**

C9orf72 overexpression in mice attenuated DSS‐induced colitis and intestinal epithelial barrier damage by stimulating ZO‐1 and Occludin expression. In LPS‐induced Caco‐2 cells, C9orf72 overexpression increased cell viability and the expression of ZO‐1 and Occludin. C9orf72 overexpression alleviated inflammation by inhibiting the cGAS‐STING pathway in colonic tissue and Caco‐2 cells.

**Conclusion:**

C9orf72 overexpression attenuated DSS‐induced colitis and intestinal epithelial barrier damage by inhibiting the cGAS‐STING pathway. C9orf72 may present a target for mitigating UC.

## Graphical Abstract

C9orf72 overexpression attenuated dextran sulfate sodium‐induced colitis and the intestinal epithelial barrier damage by inhibiting the cGAS‐STING pathway.

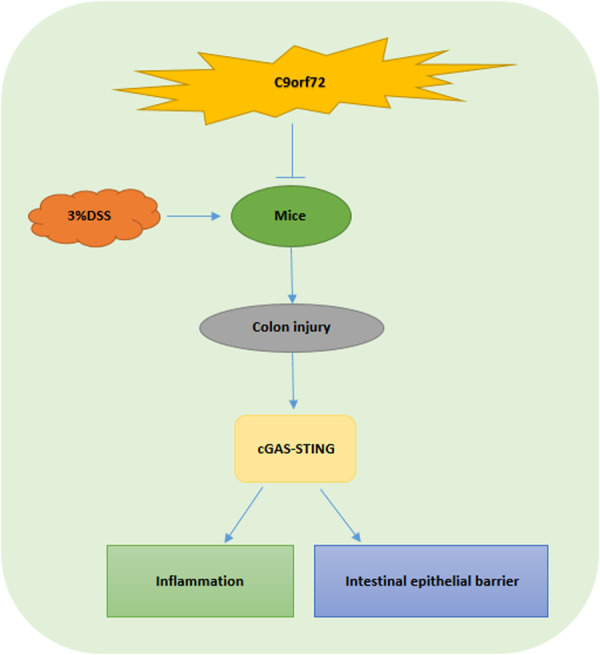

## 1 Introduction

Ulcerative colitis (UC) triggers grievous intestinal symptoms including abdominal pain, loose bowels, and bloody stools, and contributes to a high incidence of colon cancer if left untreated [1, 2]. Disturbed intestinal immune system, dysbiosis of intestinal flora, gene, and environmental factors, are widely known as the pathogenesis of UC [3]. Current treatments only manage symptoms, 10%–20% of patients still require proctocolectomy [4]. The focus of therapy is anti‐inflammatory and immunosuppressive agents, however long‐term application of these agents have exhibited manifold side effects [5]. It is urgent to optimize new targeted options for the treatment of UC.

C9orf72 mutation is associated with amyotrophic lateral sclerosis (ALS) and frontotemporal dementia (FTD). Decreased C9orf72 levels in patients with ALS/FTD cannot inhibit the inflammation mediated by stimulator of interferon genes (STING) [6]. A recent study has demonstrated that genetic correlation and shared risk loci between ALS and UC [7]. Several reports have shown that lack of C9orf72 function results in autoimmunity manifested by the enlargement of peripheral immune organs and upregulation of pro‐inflammatory cytokines [8, 9, 10]. C9orf72 decreases interleukin‐17A production in lymphoid cells [11]. Furthermore, C9orf72 inhibits gut bacteria‐induced systemic and neural inflammation [12]. C9orf72 tunes type I interferon to influence the immune system [13]. However, the effects of C9orf72 on UC are still unclear.

The Cyclic GMP‐AMP synthase (cGAS)‐STING is an innate immune signaling pathway [14]. The cGAS‐STING pathway is implicated in UC development [15, 16]. STING pathway participates in gut integrity during immune‐mediated injury [17]. Restraint of cGAS‒STING pathway could ameliorates UC [18]. However, whether C9orf72 and the cGAS‐STING pathway regulate UC by repairing the gut barrier function remains unclear.

Here, we investigated the role of C9orf72 in UC and explored its underlying mechanisms. We found that C9orf72 was observably decreased in dextran sulfate sodium (DSS)‐evoked colitis mice. Furthermore, C9orf72 overexpression could ameliorate UC by inhibiting inflammation and intestinal barrier damage via repressing the cGAS‐STING pathway. Collectively, this study displays a valuable effect of C9orf72 on UC and exerts a novel potential target for the treatment of UC.

## 2 Materials and Methods

### 2.1 Animal

The experimental protocol of our study was performed in accordance with the Guide for the Care and Use of Laboratory Animals and approved by Qingdao Municipal Hospital (Approval NO: 2023‐026, Date of review: 20230206).

Male C57BL/6J (6–8 weeks) mice were purchased from Jinan Pengyue Experimental Animal Breeding Co. Ltd. (China). DSS was purchased from Shanghai Yuanye Biotechnology Co. Ltd. (China). To detect C9orf72 expression in DSS‐treated colon tissues, mice were randomized into two groups: Control and DSS (*n* = 5). To investigate whether C9orf72 could alleviate DSS‐induced colitis in mice, mice were randomized into four groups: Control, DSS, DSS + vector, and DSS + C9orf72 (*n* = 5). Seven days before DSS treatment, mice in DSS + vector group or DSS + C9orf72 group were injected with NC lentivirus or AAV9‐C9orf72 lentivirus (5 × 10^8^ TU/mL, GeneChem, China) via the tail vein. Apart from the control group, mice were fed 3% DSS diluted in water for 7 days. The control group was treated with water for 7 days. Based on body weight loss (0–4), fecal traits (0–3), and blood in stool (0–3), the disease activity index (DAI) was scored daily as reported previously [19]. All mice were killed at Day 8. The colon length was measured.

### 2.2 Real‐Time Quantitative Polymerase Chain Reaction (RT‐qPCR)

RT‐qPCR was performed as reported previously [20]. RNA was extracted using Trizol (Invitrogen, USA). HiScript II Q Select RT SuperMix (Vazyme, China) was used to synthesize cDNA. RT‐qPCR was performed on a CFX Connect (Bio‐Rad, USA) using ChamQ SYBR qPCR Master Mix (Vazyme, China). β‐actin served as the internal reference gene. Relative levels of target genes were calculated using the 2^−ΔΔCt^ method. Primer pairs used were listed in Table 1.

**Table 1:** The primers used for RT‐qPCR.

C9orf72 mouse	Forward primer: AGCGGCGAGTGGCTATTG
	Reverse primer: TAAGCAAAGGTAGCCGCCAA
β‐actin mouse	Forward primer: CTATGCTCTCCCTCACGCCAT
	Reverse primer: ATGGCGTGAGGGAGAGCATAG
IL‐1β mouse	Forward primer: TGCCACCTTTTGACAGTGATG
	Reverse primer: TGATGTGCTGCTGCGAGATT
IL‐6 mouse	Forward primer: CCCCAATTTCCAATGCTCTCC
	Reverse primer: CGCACTAGGTTTGCCGAGTA
TNF‐α mouse	Forward primer: ACTGAACTTCGGGGTGATCG
	Reverse primer: CCACTTGGTGGTTTGTGAGTG
IL‐1β human	Forward primer: AACCTCTTCGAGGCACAAGG
	Reverse primer: GTCCTGGAAGGAGCACTTCAT
IL‐6 human	Forward primer: TCCTTCTCCACAATACCCCCA
	Reverse primer: TGTTTTCTGCCAGTGCCTCT
TNF‐α human	Forward primer: GCCCATGTTGTAGCAAACCC
	Reverse primer: TGAGGTACAGGCCCTCTGAT
β‐actin human	Forward primer: AAGGATTCCTATGTGGGCGAC
	Reverse primer: CGTACAGGGATAGCACAGCC

Abbreviation: RT‐qPCR, quantitative real‐time polymerase chain reaction.

### 2.3 Histology and Immunohistochemical Staining

The colon samples were fixed in 4% paraformaldehyde and dehydrated in graded ethanol solutions. Colon sections were stained with hematoxylin and eosin. Microscopy (Olympus BX53, Japan) was utilized to photograph the slides. The degree of lesion was scored by regions of ulceration (0–4) [21]. Immunohistochemistry was performed according to the method of a previous study [22]. Paraffin‐embedded tissue sections were deparaffinized, dehydrated, and subjected to antigen retrieval in citrate buffer. The sections were exposed to 3% hydrogen peroxide and blocked with 5% bovine serum albumin (BSA) for 1 h. Primary antibody against C9orf72 (1:2000, ab308169, Abcam, UK) was incubated at 4°C overnight before being incubated with horseradish peroxidase‐labeled Goat Anti‐Rabbit IgG (1:1000, ab6721, Abcam, UK). The color was visualized using diaminobenzidine (DAB) and hematoxylin. The samples were photographed with a light microscope.

### 2.4 Western Blot Assay

Western blot assay was performed according to the method of a previous study [23] with some modifications. Total protein was collected from colon tissues or Caco‐2 cells with radioimmunoprecipitation assay lysis (Thermo Scientific). A BCA kit (Beyotime) was used to estimate the concentration of proteins. The proteins were subjected to 10% sodium dodecyl sulfate‐polyacrylamide gel electrophoresis and transferred onto polyvinylidene difluoride membranes (Millipore). The membranes were blocked with 5% BSA in tris‐buffered saline with 0.1% Tween‐20 at room temperature for 1 h. Protein bands were detected using C9orf72 (1:2000, ab308169, Abcam), ZO‐1 (1:1000, 21773‐1‐AP, Proteintech), Occludin (1:2000, 27260‐1‐AP, Proteintech), cGAS (1:2000, 29958‐1‐AP, Proteintech), STING (1:2000, 19851‐1‐AP, Proteintech), TBK1 (1:1000, 3013, Cell Signaling Technology), Phospho‐TBK1 (1:1000, 5483, Cell Signaling Technology), and β‐actin (1:2000, 4970, Cell Signaling Technology) at 4°C overnight. Then, the membranes were incubated with secondary antibody (1:10000, ab6721, Abcam) at room temperature for 1 h. The blots were visualized by an ECL kit (Solarbio).

### 2.5 Cell Culture

Caco‐2 (Procell, China) was cultured in Minimum Essential Medium (Procell) supplemented with 20% fetal calf serum (Procell) and 1% penicillin‒streptomycin (Procell) at 37°C in a humidified 5% CO_2_. pcDNA3.1‐C9orf72 plasmid was synthesized by Tsingke Biotech (China) and transfected to Caco‐2 cells with Lipofectamine 2000. For induction of inflammation, Caco‐2 cells were treated with LPS (1 μg/mL) for 24 h. DMXAA (10 μg/mL, S1537, Selleck, USA) was added to medium for 24 h to activate STING pathway [24].

### 2.6 Cell Viability Assay

Cell viability assay was performed according to the method of a previous study [25]. Caco‐2 cells (1 × 10^3^ cells/well) were seeded into 96‐well plates for 24 h. 10 μL CCK‐8 reagent (Solarbio, China) was added to each well for 2 h. Absorbance at 450 nm was measured utilizing a microplate reader (Biotek, USA).

### 2.7 Statistical Analysis

All data are presented as mean ± SD. GraphPad Prism 8 (GraphPad, San Diego, CA, USA) was used for statistical analysis. Student's *t*‐tests was used for comparison between the two groups. Analysis of variance followed by Tukey's multiple comparisons test was used for multiple comparisons. *p* < 0.05 was considered significant.

## 3 Results

### 3.1 C9orf72 Is Declined in DSS‐Induced Colitis Mice

**Figure 1:**
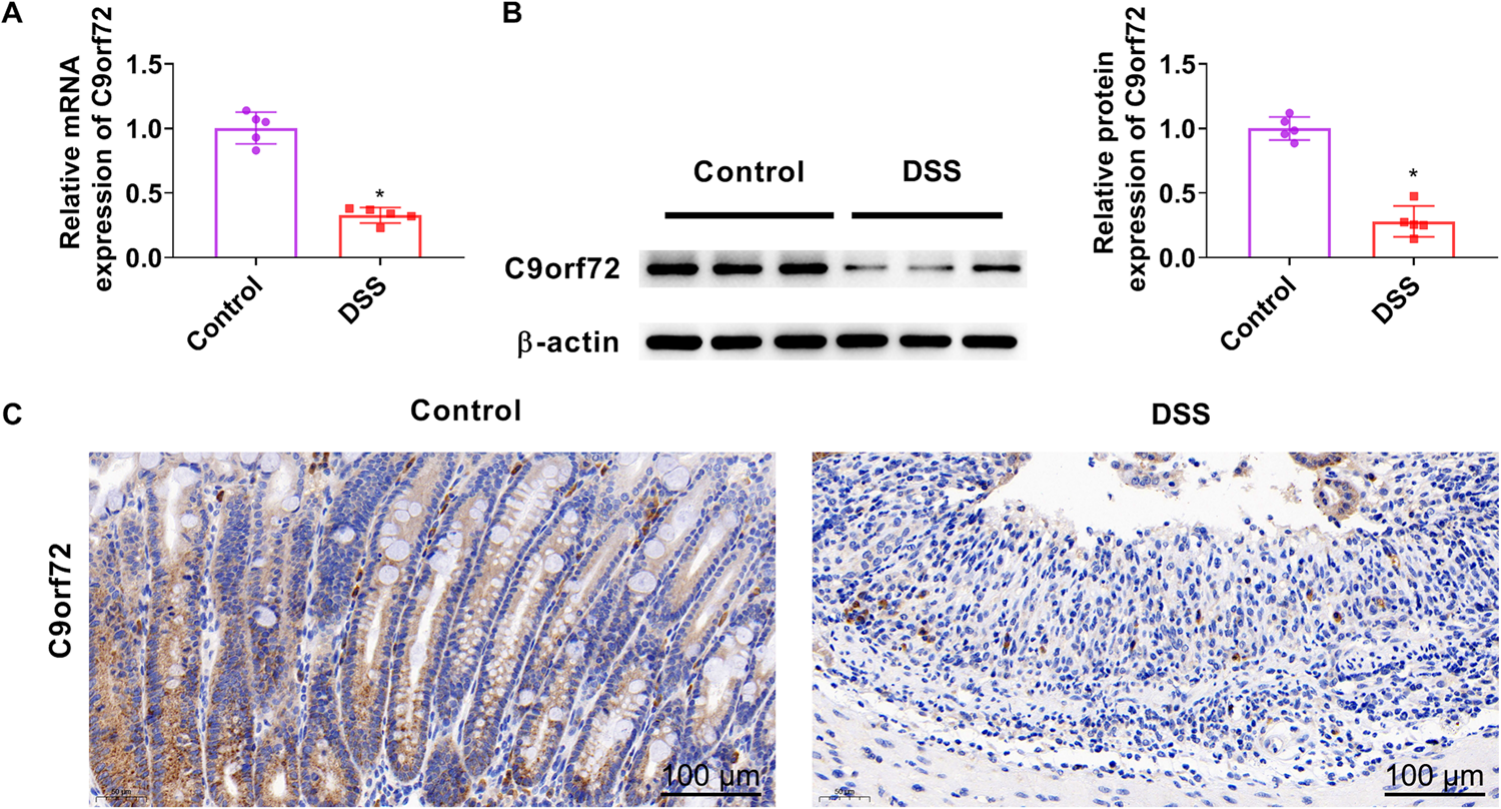
C9orf72 is declined in DSS‐induced colitis mice. Mice were received 3% DSS from Day 1 to Day 7. All mice were killed at Day 8. C9orf72 mRNA and protein expression in colon tissue of DSS‐treated mice was detected by RT‐qPCR (A), western blot analysis (B), and immunohistochemical staining (C). Bar = 100 μm. Magnification: 200. *N* = 5. Student's *t*‐tests was used for comparison between the two groups. *p < 0.05 versus control group. DSS, dextran sulfate sodium; RT‐qPCR, quantitative real‐time polymerase chain reaction.

C9orf72 expression in DSS‐treated colon tissues was detected using RT‐qPCR, western blot analysis, and immunohistochemical staining. C9orf72 expression was significantly decreased in DSS‐treated colon tissues (Figure 1A–C).

### 3.2 C9orf72 Alleviates the Histological Alterations in DSS‐Induced Colitis Mice

**Figure 2:**
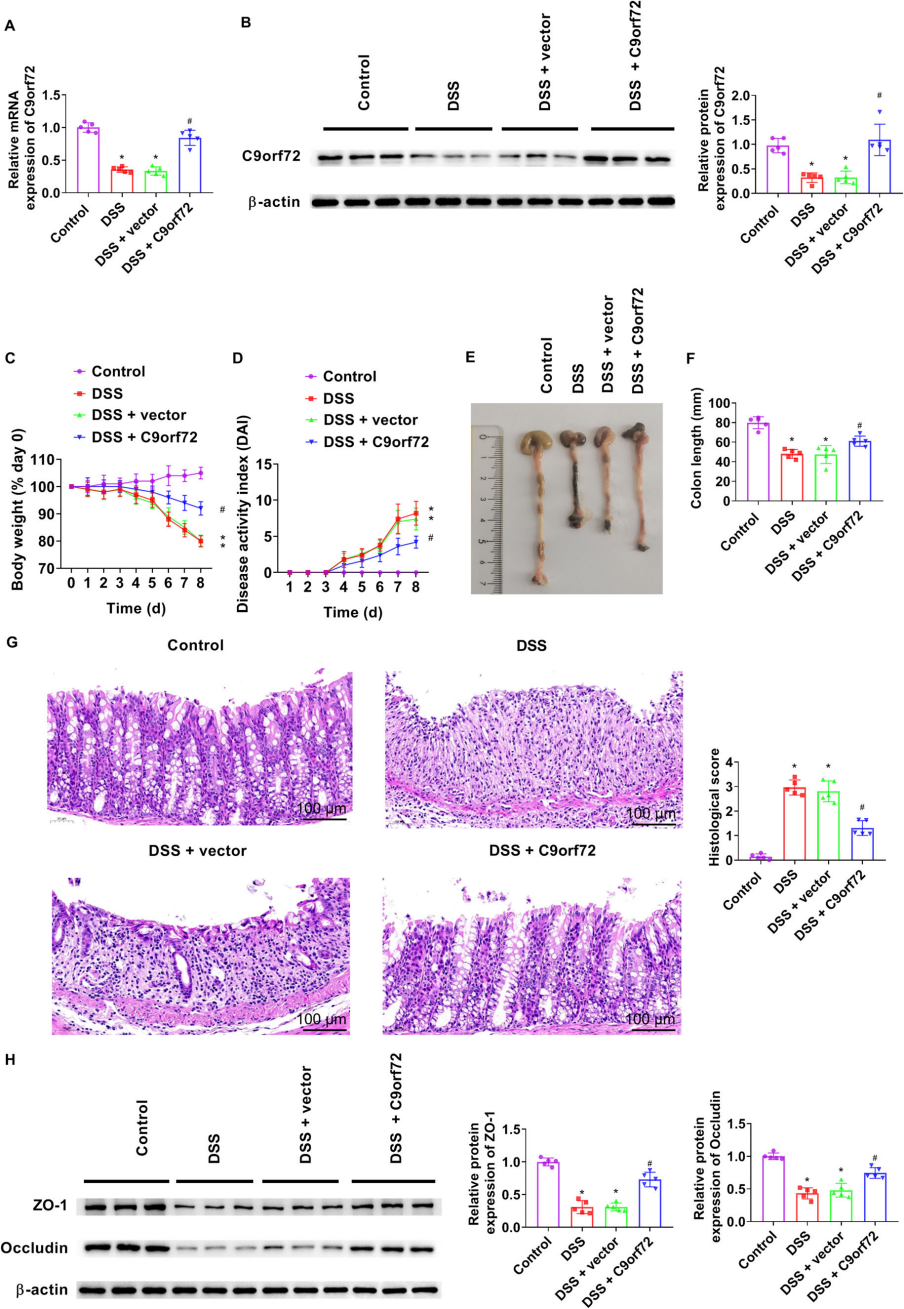
C9orf72 alleviates the histological alterations in DSS‐induced mice. Mice were injected AAV9‐C9orf72 lentivirus into the tail vein and received 3% DSS from Day 1 to Day 7. All mice were killed at Day 8. (A) C9orf72 mRNA expression in colon tissue of DSS‐treated mice was checked by RT‐qPCR. (B) Western blot analysis was performed to measure C9orf72 protein expression in colon tissues. (C) The body weight was recorded daily. (D) Daily assessment of disease activity index. (E and F) Representative colon morphology is shown and length of the colons was measured. (G) H&E staining of colon tissue sections and histological scores were quantified. Bar = 100 μm. Magnification: 200. (H) Western blot analysis was performed to measure ZO‐1 and Occludin expression in colon tissues. *N* = 5. ANOVA followed by Tukey's multiple comparisons test was used for multiple comparisons. *p < 0.05 versus control group, ^#^ *p* < 0.05 versus DSS group. ANOVA, analysis of variance; DSS, dextran sulfate sodium; H&E, hematoxylin and eosin; RT‐qPCR, quantitative real‐time polymerase chain reaction.

After overexpression with AAV9‐C9orf72, C9orf72 expression was considerably enhanced in colon tissue (Figure 2A,B). Mice in all groups except the control group, weight loss was found in DSS‐induced mice. The weight loss was significantly slower after the AAV9‐C9orf72 overexpression than DSS + vector group (Figure 2C). Mice in the DSS group displayed elevated DAI index, but DAI index greatly reduced after injection with AAV9‐C9orf72 (Figure 2D). Moreover, C9orf72 injection remarkably alleviated the decreased colon length in the DSS + vector group (Figure 2E,F). C9orf72 effectively mitigated the histological alterations in the colons of mice and increased histological score (Figure 2G). The tight junction proteins (ZO‐1 and occluding) participate in maintaining intestinal barrier [26]. The protein levels of ZO‐1 and Occludin were extensively increased in mice with colitis after C9orf72 injection (Figure 2H). These results showed that C9orf72 efficiently mitigated the severity of mice with colitis.

### 3.3 C9orf72 Reduces DSS‐Induced Colitis by Inhibiting the cGAS‐STING Pathway

**Figure 3:**
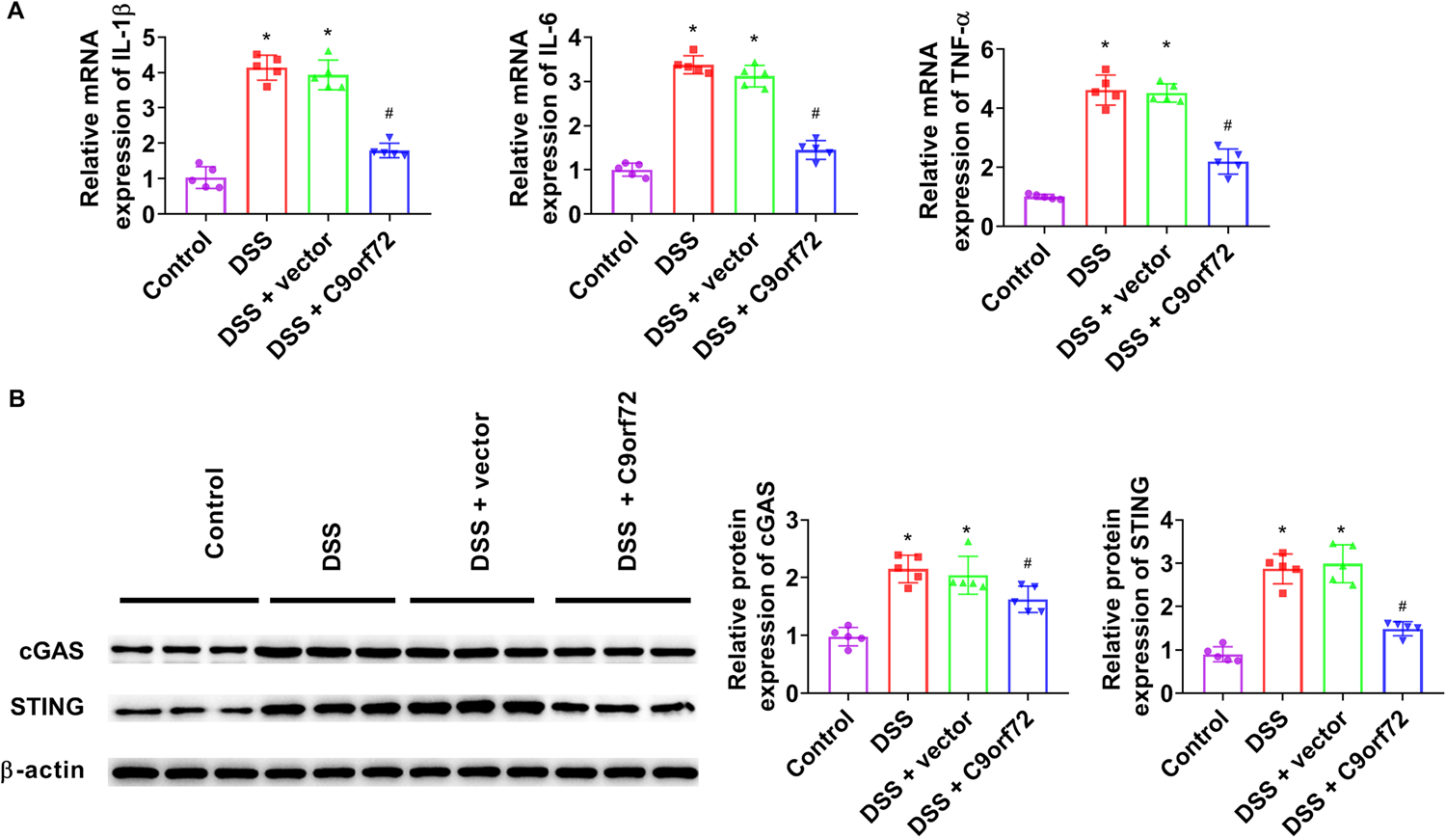
C9orf72 reduces DSS‐induced colitis by inhibiting the cGAS‐STING pathway. (A) The levels of IL‐1β, IL‐6, and TNF‐α in colon tissues were measured using RT‐qPCR. (B) Western blot analysis was performed to measure cGAS and STING expression in colon tissues. *N* = 5. ANOVA followed by Tukey's multiple comparisons test was used for multiple comparisons. *p < 0.05 versus control group, ^#^ *p* < 0.05 versus DSS group. ANOVA, analysis of variance; DSS, dextran sulfate sodium; IL, interleukin; RT‐qPCR, quantitative real‐time polymerase chain reaction; TNF‐α, tumor necrosis factor‐α.

To explore whether C9orf72 could alleviate colon inflammation by reducing colon cytokine production, we detected inflammatory cytokines. C9orf72 markedly decreased IL‐1β, IL‐6, and TNF‐α levels in the colon tissue (Figure 3A). Then, we measured the cGAS‐STING pathway‐related proteins expression using western blot analysis. C9orf72 injection significantly decreased cGAS and STING expression in DSS‐treated colon tissue (Figure 3B). These results showed that C9orf72 injection restrained the production of inflammatory cytokines in colon tissue with DSS treatment by inhibiting the cGAS‐STING pathway.

### 3.4 C9orf72 Relieves LPS‐Induced Epithelial Barrier Injury

**Figure 4:**
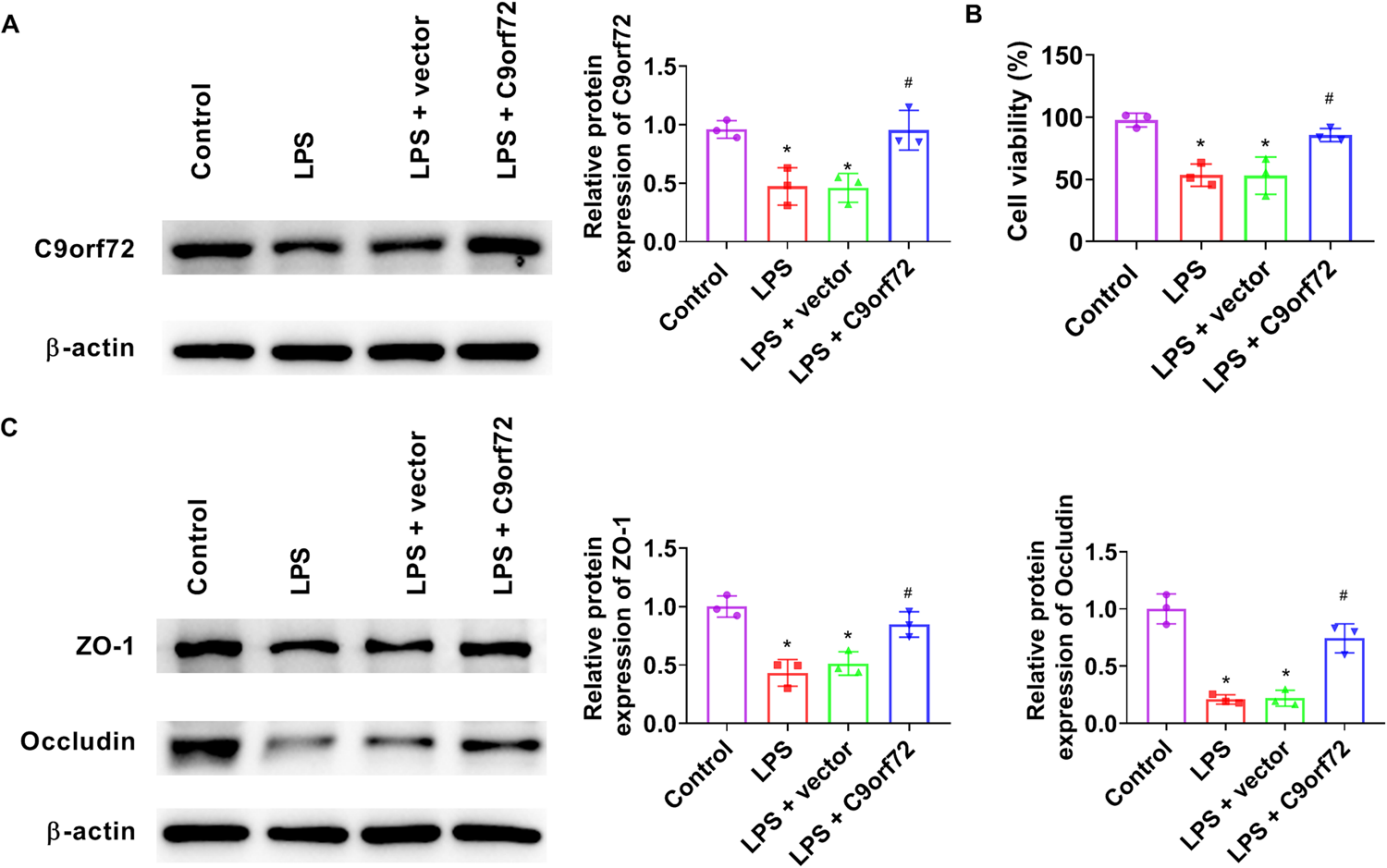
C9orf72 relieves LPS‐induced epithelial barrier injury. pcDNA3.1‐C9orf72 plasmid or empty plasmid was transfected to Caco‐2 cells. For induction of inflammation, LPS (1 μg/mL) was added to cultured cells for 24 h. (A) Western blot analysis was performed to measure C9orf72 expression in Caco‐2 cells. (B) Cell viability was detected by CCK‐8. (C) Western blot analysis was performed to measure ZO‐1 and Occludin expression in Caco‐2 cells. *N* = 3. ANOVA followed by Tukey's multiple comparisons test was used for multiple comparisons. *p < 0.05 versus control group, ^#^ *p* < 0.05 versus LPS group. ANOVA, analysis of variance; CCK‐8, Cell Counting Kit‐8; LPS, lipopolysaccharide.

pcDNA3.1‐C9orf72 plasmid was transfected to Caco‐2 cells for upregulating C9orf72 expression (Figure 4A). C9orf72 overexpression outstandingly attenuated the cell‐inhibiting effects of LPS (Figure 4B). C9orf72 overexpression significantly stimulated the expression of ZO‐1 and Occludin in LPS‐treated Caco‐2 cells (Figure 4C).

### 3.5 C9orf72 Reduces LPS‐Induced Inflammation and Epithelial Barrier Injury by Inhibiting the cGAS‐STING Pathway

**Figure 5:**
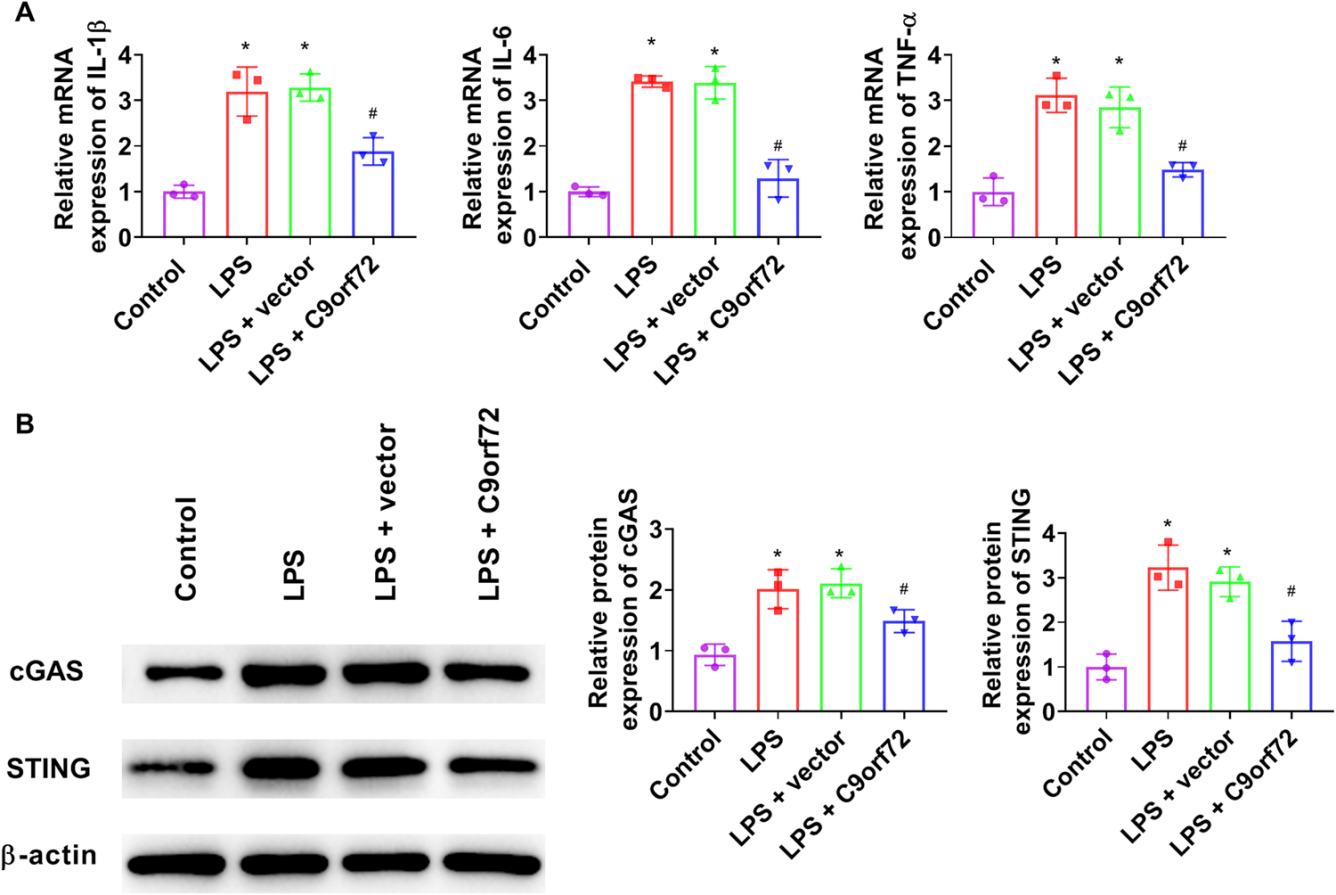
C9orf72 reduces LPS‐induced inflammation by inhibiting the cGAS‐STING pathway. (A) IL‐1β, IL‐6, and TNF‐α mRNA expression in LPS‐induced Caco‐2 cells was checked by RT‐qPCR. (B) Western blot analysis was performed to measure cGAS and STING expression in LPS‐treated Caco‐2 cells. *N* = 3. ANOVA followed by Tukey's multiple comparisons test was used for multiple comparisons. *p < 0.05 versus control group, ^#^ *p* < 0.05 versus LPS group. ANOVA, analysis of variance; IL, interleukin; LPS, lipopolysaccharide; RT‐qPCR, quantitative real‐time polymerase chain reaction; TNF‐α, tumor necrosis factor‐α.

**Figure 6:**
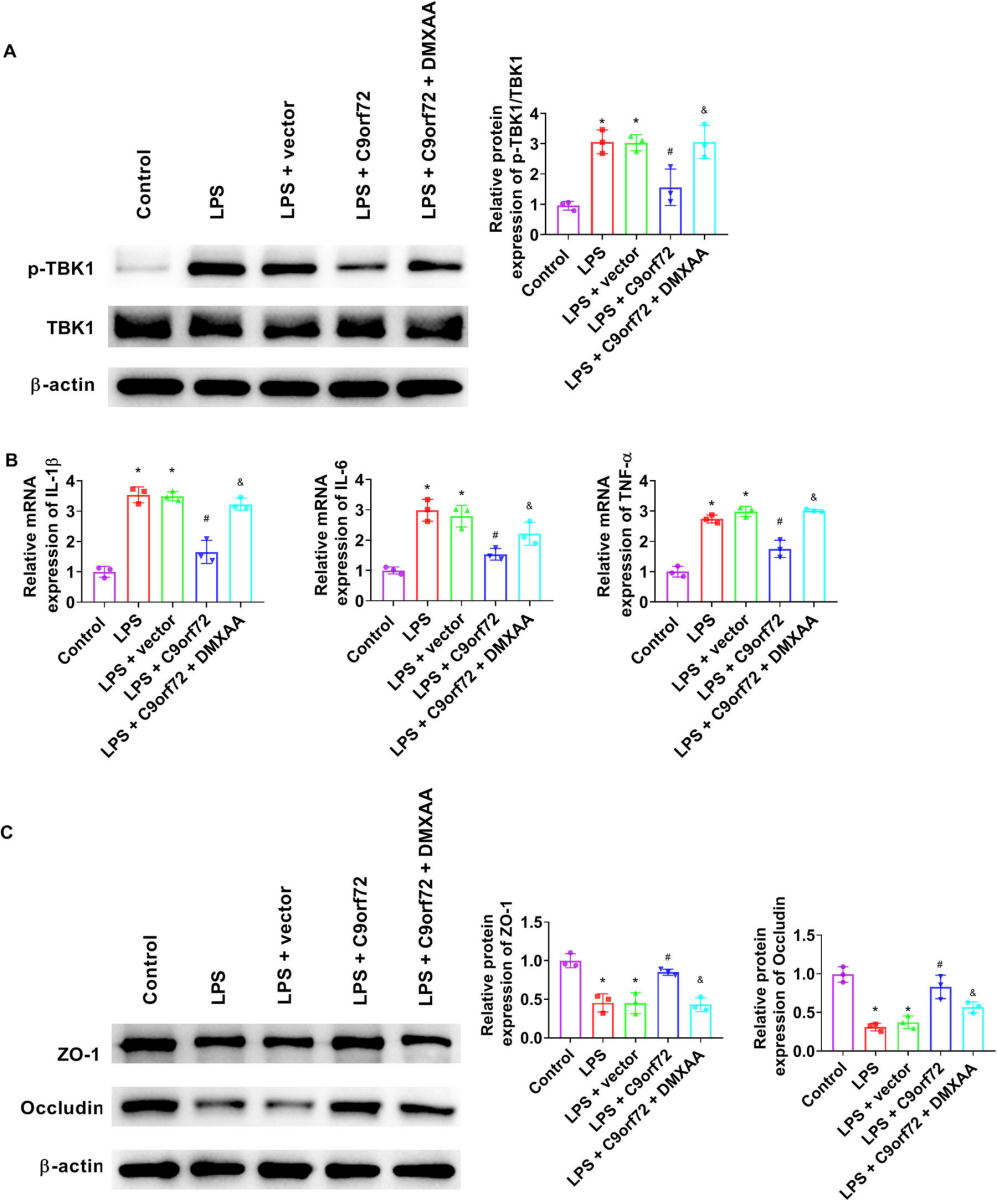
Activation of the cGAS‐STING pathway abrogates the effects of C9orf72 on LPS‐mediated inflammation and epithelial barrier injury. pcDNA3.1‐C9orf72 plasmid or empty plasmid was transfected to Caco‐2 cells. Caco‐2 cells were treated with or without LPS (1 μg/mL) and DMXAA (10 μg/mL) for 24 h. (A) Western blot analysis was performed to measure the levels of p‐TBK1in Caco‐2 cells. (B) IL‐1β, IL‐6, and TNF‐α mRNA expression in Caco‐2 cells were checked by RT‐qPCR. (C) Western blot analysis was performed to measure ZO‐1 and Occludin expression in Caco‐2 cells. *N* = 3. ANOVA followed by Tukey's multiple comparisons test was used for multiple comparisons. *p < 0.05 versus control group, ^#^ *p* < 0.05 versus LPS group, ^&^ *p* < 0.05 versus LPS + C9orf72 group. ANOVA, analysis of variance; IL, interleukin; LPS, lipopolysaccharide; RT‐qPCR, quantitative real‐time polymerase chain reaction; TNF‐α, tumor necrosis factor‐α.

C9orf72 markedly decreased IL‐1β, IL‐6, and TNF‐α mRNA levels in LPS‐treated Caco‐2 cells (Figure 5A). C9orf72 profoundly attenuated LPS‐induced cGAS and STING levels in Caco‐2 cells (Figure 5B). To clarify whether C9orf72 regulates the cGAS‐STING pathway in LPS‐treated Caco‐2 cells, a STING ligand DMXAA was used. The phosphorylation of TBK1 was observably increased upon DMXAA addition (Figure 6A). IL‐1β, IL‐6, and TNF‐α mRNA levels were substantially enhanced after DMXAA treatment (Figure 6B). After DMXAA stimulation, ZO‐1 and Occludin expression were strikingly decreased (Figure 6C).

## 4 Discussion

UC is a nonspecific chronic inflammation characterized by ulcerative lesions of the colonic mucosa [27]. The occurrence of UC is linked to integrity and permeability of intestinal barrier function [28, 29]. Nonspecific inflammation permeates the entire process of the disease [30]. Therefore, inhibiting inflammation and enhancing intestinal barrier function are key strategy for the treatment of UC.

C9ORF72 gene is identified as the most continual cause of ALS/FTD [31]. Immunoreactive microglia and enhanced inflammatory cytokines are found in patients with C9ORF72 ALS/FTD [32]. The effect of gut microbiome‐modified inflammation of ALS model is linked to C9ORF72 [33, 34]. SMCR8‐WDR41‐C9ORF72 complex regulates the autophagy‐lysosome pathway to affect inflammatory outputs in ALS/FTD [35, 36]. Smcr8 loss in mice revealed enhanced susceptibility to DSS‐induced colitis [37]. Smcr8 deficiency leads to a considerable downregulation of C9orf72 protein levels [38]. In our study, C9orf72 overexpression alleviates UC progression in DSS‐induced mice and LPS‐treated Caco‐2 cells.

DSS‐induced UC mice model is characterized by body weight loss, shortened colon length, diarrhea, and blood in stool [39]. Our results demonstrated that C9orf72 overexpression significantly reduced the disease symptoms of UC. The intestinal structure of UC mice showed destruction of the crypt structure, the disappearance of the glands, and excessive infiltration of inflammatory cells [40]. Our results indicated that C9orf72 overexpression lightened DSS‐destroyed the intestinal structures. A healthy intestinal barrier prevents hazardous substances such as toxins and pathogenic bacteria from getting into body, and maintains barrier function and intestinal immune homeostasis [41]. Inflammatory cytokines and intestinal bacteria lead to reduced expression of tight junction proteins, increased intestinal mucosal permeability, and affecting intestinal barrier function [42]. In our study, ZO‐1 and Occludin expression were enhanced in mice with colitis after C9orf72 injection. In addition, C9orf72 overexpression stimulated ZO‐1 and Occludin expression in LPS‐treated Caco‐2 cells. C9orf72 deficiency increases cytokines, chemokines, and auto‐antibodies [9]. The pro‐inflammatory factors TNF‐α, IL‐6, and IL‐1β are enhanced in UC and DSS‐induced models [43]. In our study, C9orf72 overexpression inhibited TNF‐α, IL‐6, and IL‐1β levels in DSS‐treated colon tissues.

The cGAS–STING pathway has been identified as a therapeutic target of autoinflammatory and autoimmune diseases [44]. Elevated STING expression was a characteristic of colitis [45]. In recent years, there have been many studies on inhibiting the STING pathway and alleviating colitis. For example, Chen et al. have shown that atrial natriuretic peptide relieves UC by inhibiting the cGAS‐STING Pathway [24]. Gong et al. have reported that ganciclovir administration profoundly restrains the activation of the cGAS‐STING pathway in DSS‐treated mice [15]. Gao et al. have clarified that palbociclib alleviates DSS‐induced colitis through STING [46]. There is evidence supporting that C9orf72 inhibits the STING pathway. C9orf72 deficiency increases the protein levels of STING [10]. C9orf72 restrains STING‐induced inflammation in myeloid cells [6]. In our study, C9orf72 injection significantly decreased cGAS and STING expression in the colon tissue of DSS mice and in LPS‐treated Caco‐2 cells. To clarify whether C9orf72 regulates the cGAS‐STING pathway in LPS‐treated Caco‐2 cells, DMXAA was added to Caco‐2 cells. The activation of the STING pathway simultaneously triggers TANK‐binding kinase 1 (TBK1), which induces the activation of interferon regulatory factor 3 and NF‐κB, subsequently elevating the production of type I interferon and TNF‐α [47]. In this study, the phosphorylation of TBK1 was observably increased upon DMXAA addition. DMXAA boosted DSS‐induced colonic injury and pro‐inflammatory markers [48]. Our study showed that the effects of C9orf72 on LPS‐mediated inflammation and epithelial barrier injury were reversed by DMXAA stimulation, suggesting that C9orf72 reduces inflammation and epithelial barrier injury by inhibiting the cGAS‐STING pathway.

## 5 Limitations

We only targeted the cGAS‐STING pathway to study the impact of C9orf72 expression on UC development. Other pathways involved in the treatment of UC need to be further studied. Furthermore, colorectal cancer (CRC) is one of the most serious complications of UC [49]. We will investigate whether C9orf72 could inhibit UC‐induced CRC.

## 6 Conclusion

C9orf72 overexpression attenuated DSS‐induced colitis and the intestinal epithelial barrier disorder by promoting the expression of ZO‐1 and Occludin. C9orf72 overexpression alleviated inflammation by inhibiting the cGAS‐STING pathway. C9orf72 may be a prospective therapeutic strategy for UC.

## Author Contributions

**Yue Wang**, **Ting Xu**, and **Wenjun Wang:** data curation and formal analysis.

## Ethics Statement

The experimental protocol of our study was performed in accordance with the Guide for the Care and Use of Laboratory Animals and approved by Qingdao Municipal Hospital (2023‐026).

## Consent

All authors have read and approved the manuscript.

## Conflicts of Interest

The authors declare no conflicts of interest.

## Data Availability

The data sets used and analyzed during the current study are available from the corresponding author on reasonable request.
